# Preclinical study of experimental burns treated with photobiomodulation and Human Amniotic Membrane, both isolated and associated

**DOI:** 10.1590/1518-8345.5552.3726

**Published:** 2023-03-06

**Authors:** Fernanda Cláudia Miranda Amorim, Emilia Ângela Loschiavo Arisawa, Luciana Barros Sant’anna, Ana Beatriz Mendes Rodrigues, Davidson Ribeiro Costa

**Affiliations:** 1 Centro Universitário UNINOVAFAPI, Teresina, PI, Brazil.; 2 Universidade do Vale do Paraíba, São José dos Campos, SP, Brazil; 3 Faculdade de Ciências da Saúde Pitágoras de Codó, Codó, MA, Brazil.; 4 Universidade Federal do Piauí, Teresina, PI, Brazil

**Keywords:** Burns, Amnion, Low-Level Light Therapy, Wound Healing, Skin, Rats., Queimaduras, Âmnio, Terapia com Luz de Baixa Intensidade, Cicatrização, Pele, Ratos, Quemaduras, Amnios, Terapia con Luz de Baja Intensidad, Cicatrización, Piel, Ratas

## Abstract

**Objective:**

to evaluate the effect of photobiomodulation with low-level 660 nm laser alone or associated with Human Amniotic Membrane in the repair of partial-thickness burns in rats.

**Method:**

an experimental study conducted with 48 male Wistar rats, randomized into four groups: Control, Human Amniotic Membrane, Low-Level Laser Therapy, and Low-Level Laser Therapy associated with Human Amniotic Membrane. The histopathological characteristics of the skin samples were analyzed 7 and 14 days after the burn. The data obtained were submitted to the Kolmogorov-Smirnov and Mann-Whitney tests.

**Results:**

the histological analysis of the burn injuries showed a decrease in inflammation (p<0.0001) and an increase in proliferation of fibroblasts (p<0.0001) mainly at 7 days in all treatments related to the control group. At 14 days, the greater effectiveness in accelerating the healing process was significant (p<0.0001) in the Low-Level Laser Therapy group associated with the Human Amniotic Membrane.

**Conclusion:**

the association of photobiomodulation therapies with the Human Amniotic Membrane allowed verifying a reduction in the healing process time of the experimental lesions, stimulating its proposal as a treatment protocol in partial-thickness burns.

## Resumo

**Objetivo:**

avaliar o efeito da fotobiomodulação com laser de baixa intensidade 660 nm isoladamente ou associada à membrana amniótica humana no reparo de queimaduras de espessura parcial em ratos.

**Método:**

estudo experimental com 48 ratos Wistar machos, randomizados em quatro grupos: Controle, Membrana Amniótica Humana, Terapia a Laser de Baixa Intensidade e Terapia a Laser de Baixa Intensidade associado à Membrana Amniótica Humana. As características histopatológicas das amostras de pele foram analisadas aos 7 e 14 dias após a queimadura. Os dados obtidos foram submetidos aos testes de Kolmogorov-Smirnov e Mann Whitney.

**Resultados:**

a análise histológica das lesões por queimadura mostrou a diminuição da inflamação (p <0,0001) e aumento da proliferação de fibroblastos (p <0,0001), principalmente nos 7 dias em todos os tratamentos relacionados ao grupo controle. Aos 14 dias, a maior efetividade na aceleração do processo cicatricial foi significativa (p<0,0001) no grupo Terapia a Laser de Baixa Intensidade associado à Membrana Amniótica Humana.

**Conclusão:**

a associação das terapias de fotobiomodulação à membrana amniótica humana permitiu comprovar redução no tempo do processo cicatricial das lesões experimentais, estimulando sua proposição como protocolo de tratamento em queimaduras de espessura parcial.

## Resumen

**Objetivo:**

evaluar el efecto de la fotobiomodulación con láser de baja intensidad 660 nm de sola o combinada con la membrana amniótica humana en la reparación de quemaduras de espesor parcial en ratas.

**Método:**

estudio experimental con 48 ratas Wistar macho, aleatorizadas en cuatro grupos: Control, Membrana Amniótica Humana, Terapia con Láser de Baja Intensidad y Terapia con Láser de Baja Intensidad combinada con la Membrana Amniótica Humana. Las características histopatológicas de las muestras de piel fueron analizadas a los 7 y 14 días después de la quemadura. Los datos obtenidos fueron sometidos a las pruebas de Kolmogorov-Smirnov y Mann-Whitney.

**Resultados:**

el análisis histológico de las lesiones por quemadura mostró una disminución de la inflamación (p <0,0001) y un aumento de la proliferación de fibroblastos (p <0,0001) principalmente a los 7 días en todos los tratamientos en comparación con el grupo control; a los 14 días, en el grupo de Terapia con Láser de Baja Intensidad combinada con la Membrana Amniótica Humana la mayor efectividad en la aceleración del proceso de cicatrización fue significativa (p<0,0001).

**Conclusión:**

la asociación de terapias de fotobiomodulación con la membrana amniótica humana permitió comprobar que hubo una reducción en el tiempo del proceso de cicatrización de lesiones experimentales, lo cual favorece que se proponga como protocolo de tratamiento en quemaduras de espesor parcial.

**Highlights**

(1) Association of technology with biomaterials.

(2) Innovation in Tissue Engineering.

(3) Protocol for burn treatments.

## Introduction

Skin burns are injuries caused by heat, radiation, radioactivity, electricity, friction or contact with chemical products. In the world, nearly 180,000 people die every year as a result of this problem, a reality also expressed in the last decade in Brazil by the high in-hospital mortality rate due to this cause^1^ ^,^ ^2^.

Thermal burns can occur by scalds (hot liquids), contact (hot solids) or flames^1^. In addition to being the most prevalent and strenuous, these types of lesions directly impair the phases of an adequate healing process, as they present reduced angiogenesis, sustained inflammation, oxidative stress, increased proteolysis and septicemia as main characteristics^3^.

As for depth, burns can be classified as follows: superficial-thickness (first degree), partial-superficial (second degree), deep-superficial (second degree) and full-thickness (third or fourth degree). Histologically, superficial-thickness burns only reach the epidermis; partial superficial-thickness burns reach the epidermis and papillary dermis, but the skin annexes remain intact; deep superficial-thickness burns injure the epidermis and reticular dermis and most of the skin appendages are destroyed; and, in full-thickness burns, the entire epidermis, dermis and appendages of the skin are destroyed (third degree), and may even involve the muscular fascia and/or bone (fourth degree)^4^.

The main clinical characteristics of partial superficial-thickness lesions are erythema, phlictenes, humidity, hyperemia, pressure pallor and healing time from 7 to 20 days^4^. In this sense, the healing process of burn injuries is complex, as it involves differentiated cells that are activated during the different and overlapping phases of the tissue repair process called inflammation, proliferation and remodeling^5^.

The injuries resulting from burns establish challenges in the skin repair process, as the burned area presents characteristics that hinder repair, such as irregular edges and tissue necrosis, in addition to being capable of reaching the epidermis, dermis and deep tissues. The need for hospitalizations and high hospital costs is also highlighted^6^.

The multifaceted environment of burn wound healing has stimulated the investigation of innovative therapeutic interventions that enable immediate repair of this problem^7^. Thus, defining an appropriate strategy in view of the needs and complexity of burns becomes fundamental for the success of therapeutic treatments in terms of performance and cost. In this sense, biomaterials and new technologies stand out for having general properties capable of inducing different biological responses that can be adapted according to the application^8^.

In the context of technologies, photobiomodulation with the use of low-level laser therapy (LLLT) has stood out for favoring wound healing due to its biomodular effects^9^ ^-^ ^11^. In contrast, related to biomaterials, the Human Amniotic Membrane (HAM) has been used as a promising alternative, with great potential for application in regenerative medicine for presenting low antigenicity and protection against infections, as well as for acting as a substrate for epithelization^12^. Therefore, several studies have clinically evaluated the benefit of HAM as a biological substitute^13^ ^-^ ^15^.

Thus, considering the complexity of burn therapy and the need for experimental studies that investigate alternative treatments that favor tissue regeneration in this condition, this study evaluated the effect of LLLT associated with HAM in the repair of superficial partial thickness burns in rats.

## Method

### Type of study

This is an experimental research study with a quantitative approach.

### Study locus

The research was conducted at the experimental surgery laboratory of the UNINOVAFAPI University Center, located in the municipality of Teresina (PI), Brazil. 

### Study period

Data collection took place from January to March 2019.

### Animals

A total of 48 male rats (*Rattus norvegicus albinus,* Wistar) were studied: 40 days old, weighing 200 ± 50 g, kept in polypropylene cages under aseptic conditions, specific feed with food and water *ad libitum*, and exposed to a 12/12-hour light-dark cycle, housed in individual cages.

### Study groups

The animals were randomized and allocated into four groups with twelve animals each, namely: Control (C); animals subjected to experimental burns without treatment; Human Amniotic Membrane (HAM), rats subjected to experimental burns treated with application of HAM fragment; Low-Level Laser Therapy (LLLT), animals subjected to experimental burns treated with LLLT; and LLLT+HAM, animals subjected to experimental burns treated with the association of LLLT and HAM. The animals from all 4 (four) groups were subdivided into 2 (two) subgroups according to experimental times of 7 and 14 days, containing 6 animals in each.

### Data collection

The experimental protocol was developed in five stages: capture of the human placenta, processing of the biomaterial, induction of burns, and application of HAM fragments and LLLT, isolated or associated.

The placentas were collected from two selected parturients, after signing the Free and Informed Consent Form (FICF), subjected to elective cesarean section, with a healthy clinical history, negative serological tests for HIV-1, VDRL, HbsAg and anti-HCV, and gestational age from 37 weeks to 41 weeks and 6 days (full term placenta), according to criteria established in a previous study^16^. 

The placentas were inspected immediately after removal, placed in a sterile plastic bag, packed at a temperature of 10ºC and transported to the experimental surgery laboratory. The biomaterial was processed in an aseptic environment following the protocols described^16^, isolating the HAM that was sectioned into fragments of suitable dimensions for this research (4 x 4 cm) that were used in 24 hours^17^. 

Initially, the animals were weighed, sedated (Xylazine 2%, 0.01 mL/kg and Ketamine 10%, 0.005 mL/kg) and their dorsal region was epilated. The experimental burn was induced using a beaker (3 cm in diameter), filled with 50 mL of water heated to 100°C, supported in direct contact with the shaved region skin for 10 seconds, without additional pressure. Subsequently, the lesions were evaluated considering the macroscopic aspects, which included observation of staining (red or pink) and presence of a bubble to characterize the superficial partial thickness burn^4^.

The animals from group C received no treatment. In the animals from the HAM and LLLT+HAM groups, HAM fragments were applied immediately after the burn, always with the mesenchymal face in contact with the skin lesion area, exceeding its edges by 1 cm, and fixed with a topical adhesive. 

The protocol used in the animals from the LLLT group included laser application. The first applications with laser occurred 30 minutes after burn induction and were repeated at 24-hour intervals. A Laserpulse Ibramed^©^ device (*Indústria Brasileira de Equipamentos Médicos* - IBRAMED) was used, and the irradiation parameters emnployed in the experiment were the following: wavelength of 660 nm, power of 30 mW, with an irradiation time of 12 seconds *per* point, contact area of 0.06310 cm^2^, energy density of 6 J/cm^2^, continuous pulse parameters, with treatment in a single dose, in a 24-hour interval, and with the animal’s back as anatomical location.

The irradiations were applied punctually at four equidistant points, in the shape of a cross, 1 cm between the edge of the lesion and the irradiation point, with a 90º angle and protection of the laser tip with a sterile transparent film, avoiding possible contamination.

In the HAM+LLLT group, the protocols described for the HAM and LLLT groups were associated, and the laser was applied on the amniotic membrane every 24 hours at both experimental times studied (7 and 14 days).

The animals were euthanized according to the experimental time studied (7 or 14 days) with administration of an overdose of anesthetic (sodium thiopental 100 mg/kg, intraperitoneally). The burned area and surrounding tissue were carefully removed and fixed in neutral buffered formalin (10%). 

### Histological techniques 

The burned skin area and the surrounding area, including the entire area of the lesion and the edges of adjacent normal tissue (1 cm from the edge), were removed and fixed in 4% buffered formalin for 48 hours and then transferred to a 70% alcohol solution, cleared with xylene and embedded in paraffin. Four longitudinal semi-serial histological sections of 2 to 3 μm were made from each block, spread on glass slides and stained with Hematoxylin and Eosin (H&E) and Picrossirius Red. 

### Histological and morphometric assessments 

The injured area was evaluated macroscopically after the burn and throughout the experiment considering skin color, presence of blisters and superficial crust formation. The histological sections stained with H&E were scanned using a Leica^®^ DM 2500 microscope coupled to a Leica^®^ DFC 425 camera and the Leica^®^ Application Suite LAS v3.7 program. The images were obtained from the cross sections of four sequential fields of each slide, with the 10X and 40X objectives under a light microscope. To quantify the number of inflammatory cells (neutrophils and macrophages) and fibroblasts (young and adult), the images were analyzed using the ImageJ software, which allowed elaborating a grid and the individual marking of the cell nuclei with the aid of the manual counting tool. 

The slides stained with Picrosirius Red were evaluated by digital image analysis to calculate the area occupied by the deposition of collagen types I and III, and photographed with the 10X objective, with a polarized light microscope (Leica^®^ DM 2000) coupled to the camera (Leica^®^ DFC 425). The Image-Pro Plus 4.5 program was used to quantify the percentage of type I and III collagens. When analyzed in association with polarized light, presence of collagen considered the following specifications for identification of the collagen types: collagen type I - yellow-reddish color; and collagen type III - green-whitish color. All histomorphometric analyses were performed blindly. 

### Statistical analysis 

The data collected were evaluated for the coefficient of variation and sample distribution for determination of the statistical test. The GraphpadPrism V program (GraphPad Software, California, USA) and the Kolmogorov-Smirnov test were used to analyze data distribution. Due to the non-parametric presentation, the Mann-Whitney test was applied in the intragroup analysis. For the comparison between groups, the Kruskal-Wallis test was applied with Dunn’s Multiple Comparison Test powders (multiple comparisons - intergroup analysis). A 95% confidence interval and a 5% significance level (p<0.05) were considered. The data are presented as mean ± standard error (of the mean).

### Ethical aspects

This study was approved by the Research Ethics Committee of the University of Vale do Paraíba (2.077.418) for the use of HAM and by the Ethics Committee on Animal Experimentation of the UNINOVAFAPI University Center with No. 005P/V2/2017, following the recommendations proposed by Resolution 446/2012 of the National Health Council (*Conselho Nacional de Saúde*, CNS), 

## Results

**Figure 1:**
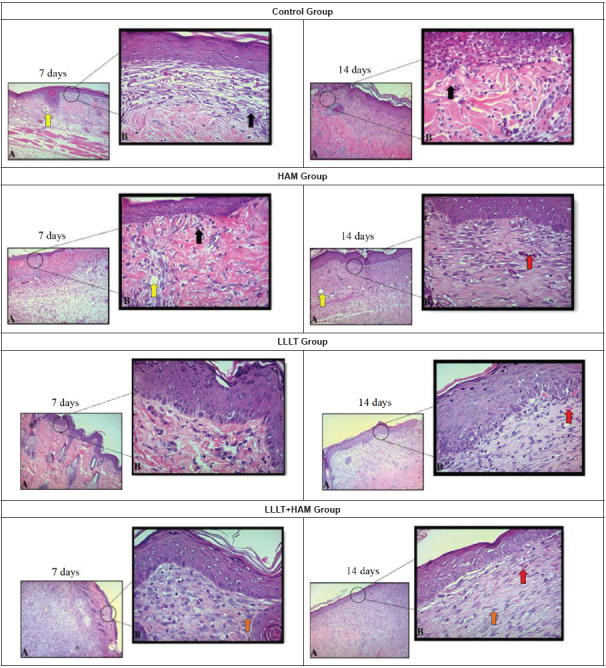
Photomicrographs showing histopathological changes in partial superficial-thickness burns in rats from groups C, LLLT, HAM and LLLT+HAM at seven and fourteen days. Teresina, PI, Brazil, 2022

The evaluation of the photomicrographs of the histological slides stained with H&E shows progression of wound healing after partial superficial-thickness burns in all groups and experimental times (Figure 1).

Lower A (10X) and upper B (40X) magnification. The red arrows represent the blood vessels, the yellow arrows are edema area, the black arrows show inflammatory infiltrates and the orange arrows are fibroblasts.

**Figure 2:**
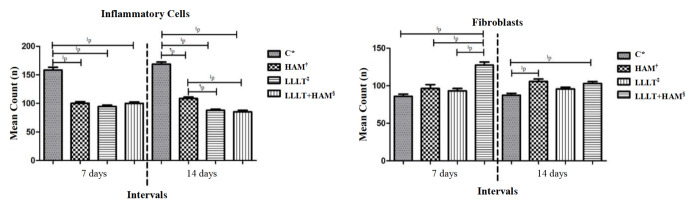
Effect of photobiomodulation with continuous LLLT (660 nm) applied alone and in association with HAM in the mean count of fibroblasts and inflammatory cells in partial superficial-thickness burns. Teresina, PI, Brazil, 2022

Use of LLLT associated with HAM significantly decreased the number of inflammatory cells when compared to the other treatment protocols. In the intergroup analysis, it was evidenced that group C presented the highest mean number of inflammatory cells in the periods analyzed. At 14 days there was a statistical difference in the animals from the LLLT+HAM group in relation to the C and HAM groups, with emphasis on the reduction in the number of inflammatory cells in the LLLT+HAM animals (Figure 2). 

There was an increase in the number of fibroblasts in the LLLT+HAM group when compared to the C, HAM and LLLT groups in the experimental time of 7 days. At day 14, the animals from the LLLT+HAM and HAM groups presented higher means when compared to the other groups (Figure 2). 

Kruskal-Wallis test applied with Dunn’s Multiple Comparison Test powders (multiple comparisons - intergroup analysis). *C = Control Group; ^†^HAM = Human Amniotic Membrane Group; ^‡^LLLT = Low-Level Laser Therapy Group; ^§^LLLT+HAM = Low-Level Laser Therapy Group associated with Human Amniotic Membrane; ^║^p = Extremely significant difference (*p*<0.0001); ^¶^p = Significant difference (*p*<0.05)

The intragroup analysis of the inflammatory cells showed that only the LLLT and LLLT+HAM groups evidenced statistically significant differences. In relation to the mean of fibroblasts, statistically significant values were observed in the HAM and LLLT+HAM groups (Table 1).

**Table 1:** Intragroup histopathological analysis of the mean Inflammatory Cell and Fibroblast count (mean ± standard error) after treatment with LLLT (660 nm), HAM and combination of both therapies (LLLT+HAM) 7 and 14 days after partial surface thickness thermal burns in rats (n=48). Teresina, PI, Brazil, 2022

Groups	Inflammatory cells			Fibroblasts		
	Experimental times			Experimental times		
	7 days	14 days	p*	7 days	14 days	p*
Control	*158.4 ± 4.92*	*168.8 ± 3.64*	*ns* ^†^	*85.8 ± 3.05*	*87.5 ± 2.42*	*ns* ^†^
HAM	*100.5 ± 2.53*	*108.8 ± 2.40*	*ns* ^†^	*96.2 ± 6.23*	*105.8 ± 5.31*	p^‡^
LLLT	*94.6 ± 2.43*	*87.5 ± 2.12*	p^‡^	*102.9 ± 2.56*	*97.04 ± 2.82*	*ns* ^†^
LLLT+HAM	*99.42 ± 2.451*	*85.24 ± 2.64*	p^‡^	*134.4 ± 3.30*	*93.21 ± 3.04*	p^§^

Mann-Whitney test applied for intragroup analysis. *p = Significance level; ^†^ns = Non-significant difference; ^‡^p = Significant difference (p<0.05); ^§^p = Extremely significant difference (p<0.0001).

**Figure 3:**
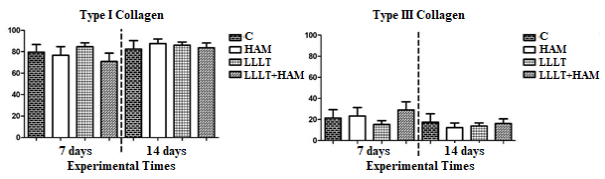
Graphical representation of the effect of photobiomodulation with LLLT and amniotic membrane, applied alone or in combination, on the percentage of collagen types III and I in partial superficial-thickness burns in Wistar rats (n=48). Teresina, PI, Brazil, 2022

The percentage of collagen types I and III (%) in the periods analyzed did not differ across the experimental groups. However, when compared to group C, there was a slight increase in type III collagen in the LLLT+HAM group on the seventh day, although without statistical significance (Figure 3). 

Kruskal-Wallis test applied with Dunn’s Multiple Comparison Test powders (multiple comparisons - intergroup analysis).

**Figure 4:**
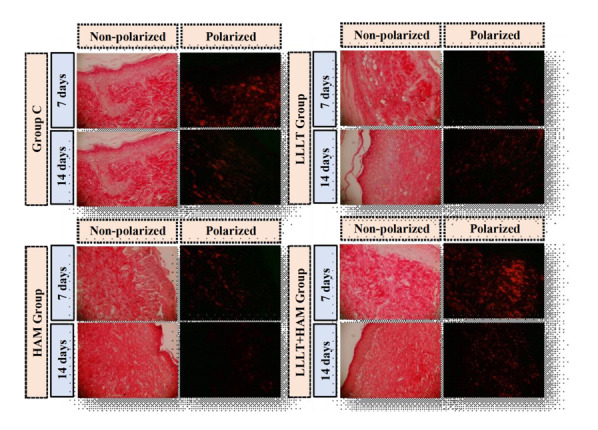
Photomicrographs observed with polarized and non-polarized light showing type I and III collagen fibers in partial superficial-thickness burns in Wistar rats from groups C, HAM, LLLT and LLLT+HAM at seven and fourteen days, 10X objective (n=48). Teresina, PI, Brazil, 2022

The evaluation of the photomicrographs of the histological slides stained with picrosirius showed type I and III collagen fibers of the lesions after partial superficial burns in all groups and experimental times (Figure 4). 

## Discussion

Using rats in experimental research involving tissue repair in burns has been a frequent practice, especially because their skin composition (epidermis and dermis) is similar to that of human skin, in addition to presenting low cost and reduced healing times. However, morphology of the rodent skin is unique and differs from the architecture of the human skin because it presents low adherence to the underlying structures, presence of the l-gluconolactone enzyme that converts l-gluconogamalactone into vitamin C, wound contraction healing and lower risk of infection^18^.

This study investigates the association of photobiomodulation (LLLT) with HAM for the treatment of experimentally induced partial superficial burn injuries in rats. It evaluated tissue repair in these lesions and describes the evolution of the healing process using isolated and combined therapies at experimental times of 7 and 14 days.

Published clinical and experimental studies have used LLLT for the healing of acute and chronic wounds; however, there are few reports of the use of this technology combined with other therapies in partial superficial burns and no study using HAM as an adjunct in treatment^7^ ^,^ ^10^ ^,^ ^11^ ^,^ ^19^.

In our research it was verified that, at the experimental times, there was a significant reduction of inflammatory cells in all treated groups in relation to the control group. However, it was found that, although the therapies are effective in reducing inflammation, the combination of the LLLT+HAM therapies was more effective than their isolated use, evidencing the additive effects on modulation of the inflammatory activity that the combined treatment can promote. Considering that the anti-inflammatory effects of the isolated use of these therapies in burns are already described in the literature, our findings evidence that their combined use enhances the inflammatory activity. These findings also suggest that the combined therapy acts harmoniously, that HAM would function as a biological substrate and that it would have its action enhanced from the microenvironment conducive to cell oxygenation, growth and modulation created with the irradiated light.

We discovered that all therapies (both isolated and associated) were effective in reducing inflammatory cells at both experimental times; such findings reveal the importance of their early use so that the tissue repair process occurs without delays. In this sense, a study with induced acute wounds revealed that exacerbated and prolonged inflammation causes harms in the re-epithelialization process by modifying the formation of granulation tissue, with an increase in the possibility of scar formation^20^.

It is worth noting that, in the intergroup analysis of the second experimental period, LLLT+HAM was extremely effective when compared to the isolated therapies. In addition to that, LLLT alone was more effective in reducing the mean number of inflammatory cells than the treatment of burns with HAM alone.

Photobiomodulation with LLLT has been used to reduce inflammation, pain and edema, as well as to preserve and restore tissues damaged by the injury. These effects can be achieved using wavelengths between 600 and 1000 nm. In this sense, clinical and experimental studies with partial and total thickness burns have used photobiomodulation with LLLT with a wavelength of 660 nm^21^, ratifying choice of this parameter also in our study ^(^5^, ^19^, ^22^, ^23^)^.

Similarly to the results found in our study, modulation of the inflammatory response was evidenced in the healing of skin grafts in rats in a recent study that used the same LLLT irradiation protocol^24^. In addition to that and corroborating our findings, the effects with a single LLLT dose have been pointed out in the literature, with acceleration of the inflammatory phase in skin repair among them^22^.

It is known that inflammation and angiogenesis are important factors in determining wound healing and that the decrease in inflammation enables an increase in angiogenesis. Thus, the modulation properties of the inflammation and cell proliferation levels are found in research studies with LLLT^25^ ^,^ ^26^.

In the context of the association of LLLT with other therapies in burn treatments, a study that used this tool combined with medicinal honey obtained results for inflammation and pain attenuation in burn healing and acceleration of the repair process characterized by increased cell proliferation^7^, corroborating the same effects of the therapeutic association protocol used in our study.

It is noted that, for the treatment of superficial partial thickness burns, the ideal dressings are those that can preserve heat, provide moisture, avoid contamination by microorganisms, be safe and not adhere to the injury or require frequent exchanges^27^. Therefore, HAM stands out for being a biomaterial that has all the listed characteristics^12^.

HAM has been applied to acute and chronic wounds, as evidenced by the promising results obtained with the application of this biomaterial in the healing of these lesions due to its properties^28^ ^,^ ^29^. It is noted that the dressings used with this biomaterial or in association with other products can facilitate proliferation of fibroblasts and contribute to the release of angiogenic factors^30^. 

In the intergroup analysis context and regarding proliferation of fibroblasts, our results show that LLLT+HAM was effective at both experimental times. It is noted that fibroplasia was benefited by the use of the combined therapies since, already in the initial phase of the experiment, HAM may have modulated the LLLT action, enhancing the cell activation process and, consequently, culminating in early onset of the proliferative phase, characterized by an extremely significant increase of fibroblasts in relation to all other experimental groups. This fact evidences the high capacity to repair the burned tissue when choosing the treatment with the associated therapies. 

A number of studies evidence a biological response favoring the tissue repair process by stimulating proliferation of fibroblasts, and the improvement in microcirculation has also been proven in the context of photobiomodulation with LLLT^31^ ^-^ ^33^. 

The data obtained in our study reinforce the beneficial properties of using LLLT and HAM reported in previous studies in the context of the isolated use of these therapies or their association with other products^7^ ^,^ ^30^ ^-^ ^34^. However, we showed that the combination of therapies (LLLT and HAM) in burns can yield excellent results due to the sum of the modulating and protective effects in the different tissue repair phases.

Considering the complexity of the healing process in burns, our results are presented as a preclinical phase that encourages expansion of the techniques for evaluating the effects determined by the association of LLLT and HAM, in order to detect more information about the interaction of photobiomodulation with the biomaterial, aiming at a future introduction of this combination therapy in clinical protocols for the treatment of superficial partial thickness burns. 

In our study, there was no statistical significance for the percentage of collagen types I and III in the periods analyzed, although in the photomicrographs there was progression in the cell organization process evidenced in the treatment groups. In this sense, we suggest extending the experimental time to contemplate all the dynamics of the repair process. Furthermore, non-measurement of the lesion is pointed out as a study limitation, limiting wound contraction monitoring. In addition to that, immunological markers were not used, which are important evaluators of the tissue and biochemical reactions in the tissue-related repair. 

## Conclusion

Our study showed that photobiomodulation with low-level 660 nm laser acts synergistically to the topical application of the Human Amniotic Membrane, constituting an effective therapeutic protocol in the treatment of superficial partial thickness burns. Combination of the therapies enhanced the anti-inflammatory effects and stimulation of cell proliferation, accelerating the tissue repair process.
